# DZNep inhibits H3K27me3 deposition and delays retinal degeneration in the *rd1* mice

**DOI:** 10.1038/s41419-018-0349-8

**Published:** 2018-02-22

**Authors:** Shijie Zheng, Lirong Xiao, Yu Liu, Yujiao Wang, Lin Cheng, Junjun Zhang, Naihong Yan, Danian Chen

**Affiliations:** 1 Research Laboratory of Ophthalmology and Vision Sciences, Torsten-Wiesel Research Institute of World Eye Organization, State Key Laboratory of Biotherapy, West China Hospital, Sichuan University, 610041 Chengdu, China; 2 Department of Ophthalmology, West China Hospital, Sichuan University, 610041 Chengdu, China; 3 Program in Systems Biology, University of Massachusetts Medical School, 368 Plantations Street, Worcester, MA 01606 USA; 4 Shenzhen Key Laboratory of Ophthalmology, Shenzhen Eye Hospital Affiliated to Jinan University, 518040 Shenzhen, China

## Abstract

Retinitis pigmentosa (RP) is a group of inherited retinal degenerative diseases causing progressive loss of photoreceptors. Numerous gene mutations are identified to be related with RP, but epigenetic modifications may also be involved in the pathogenesis. Previous studies suggested that both DNA methylation and histone acetylation regulate photoreceptor cell death in RP mouse models. However, the role of histone methylation in RP has never been investigated. In this study, we found that trimethylation of several lysine sites of histone H3, including lysine 27 (H3K27me3), increased in the retinas of *rd1* mice. Histone methylation inhibitor DZNep significantly reduced the calpain activity, delayed the photoreceptor loss, and improved ERG response of *rd1* retina. RNA-sequencing indicated that DZNep synergistically acts on several molecular pathways that regulate photoreceptor survival in *rd1* retina, including PI3K-Akt and photoreceptor differentiation pathways, revealing the therapeutic potential of DZNep for RP treatment. PI3K-Akt pathway and H3K27me3 form a feedback loop in *rd1* retina, thus PI3K inhibitor LY294002 reduces phosphorylation of Ezh2 at serine 21 and enhances H3K27me3 deposition, and inhibiting H3K27me3 by DZNep can activate PI3K-Akt pathway by de-repressing gene expression of PI3K subunits *Pik3r1* and *Pik3r3*. These findings suggest that histone methylation, especially H3K27me3 deposition is a novel mechanism and therapeutic target for retinal degenerative diseases, similar to H3K27me3-mediated ataxia-telangiectasia in *Atm*^*−/−*^ mouse.

## Introduction

Retinitis pigmentosa (RP) is a group of inherited retinal degenerative diseases characterized by a two-stage process, in which the rod photoreceptors degenerate first, followed by cone cell degeneration^1, 2^. There are more than 1.5 million RP patients worldwide^3, 4^. Unfortunately, there has been no effective therapy available for RP right now. *Rd1* mice, one of the most commonly used RP animal model, carry a loss-of-function mutation in the rod-specific *Pde6β* gene, representing a subset of RP patients^3, 5, 6^. Several mechanisms are involved in the pathogenesis of *rd1* retina, including apoptotic and non-apoptotic cell death, cell-intrinsic factors, disrupted intracellular Ca^2+^ homeostasis, and epigenetic modifications^7–11^.

Epigenetic modifications, including DNA methylation, histone acetylation, and methylation, affect gene expression but do not change DNA sequences. Recent studies have also identified histone variants, microRNAs, and long non-coding RNAs as critical epigenetic mechanisms^12, 13^. Epigenetic deregulation has been involved in a variety of retinal diseases, including retinal fibrosis, retinoblastoma, RP, age-related macular degeneration, and diabetic retinopathy^14–18^. *Rd1* retinas have high levels of 5-methyl cytosine^11^, inhibiting DNA methyltransferases (DNMTs) in *rd1* retinal explants can reduce photoreceptor death^19^. Histone deacetylase-4 (HDAC4, class II HDAC) overexpression prolongs rod survival in *rd1* mice^15^. However excessive HDAC activation can also induce photoreceptor degeneration, as inhibition of HDACs I/II activity in *rd1* retinal explants strongly prevents photoreceptor death^10^.

Histone methylation is catalyzed by histone methyltransferases (HMT), and can activate or suppress transcription^20–22^. It has been reported that histone lysine methylation is associated with retinal development^23, 24^. Previously we reported that knocking out *Bmi1* results in extensive photoreceptor survival in *rd1* retina^25^. *Bmi1* is a component of the polycomb repressive complex 1 (PRC1), which compacts chromatin and represses gene expression^26^. We also reported that knocking out *Ezh2* in embryonic retinal progenitors results in progressive photoreceptor degeneration^27^. Ezh2 is the core component of the PRC2 and catalyzes the addition of methyl groups to histone H3 at lysine 27 (H3K27), leading to epigenetic silencing^28^. Our results indicate that both PRC1 and PRC2 may play critical roles in RP pathogenesis. Ezh2 can also recruit DNMT3 which is upregulated in *rd1* retina, thus providing a direct link between two key epigenetic repression systems (PRC2 and DNA methylation) in *rd1* retina^19, 29, 30^.

Since Ezh2 is mainly expressed in embryonic retina^24, 27, 31^, we examined whether histone lysine methylation changes in postnatal *rd1* retinas. To our surprise, we found that postnatal *rd1* retina has higher histone lysine trimethylation, and HMT inhibitor 3-deazaneplanocin A (DZNep) actually delays the retinal degeneration. This result is similar to the H3K27me3-mediated neurodegeneration and ataxia-telangiectasia in *Atm*^*−/−*^ mouse^32^, suggesting that HMT inhibition in wild type (*wt)* retinal progenitors and postnatal *rd1* retinal cells may have opposite effects on photoreceptor survival. Our study discovers a novel strategy to treat retinal degeneration.

## Results

### *Rd1* retina has high levels of histone H3 trimethylation including H3K27me3

**Fig. 1:**
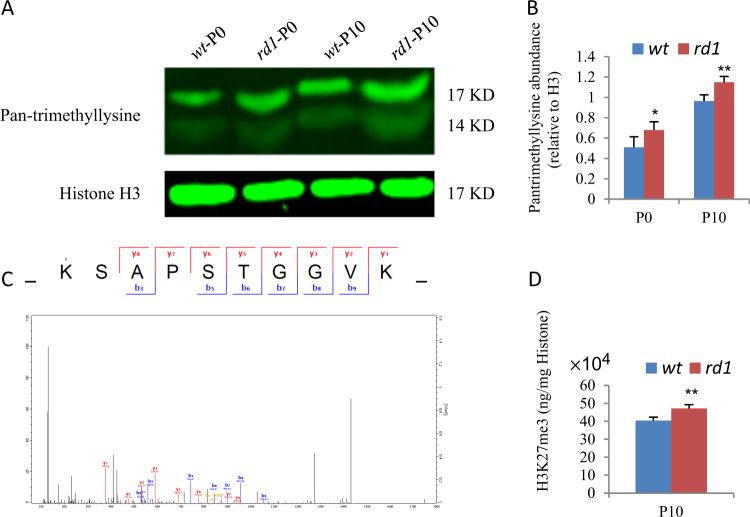
Expression of pan-trimethyllysine and H3K27me3 in *wt* and *rd1* retina at P0 and P10. **a** Representative Western blots of indicated protein of indicated age and genotypes. **b** The quantification of pan-trimethyllysine levels relative to histone H3 of **a**. **c** High-resolution MS/MS spectrum of H3K27me3 of P10 retinas. **d** The expression of global H3K27me3 in *rd1* and *wt* retinas at P10. Error bars represent SD of measurements from at least three animals and asterisks indicate significant differences between retinas of *wt* and *rd1* mice (*p < 0.05, **p < 0.01, *t*-test)

Trimethylation of lysine residues of histone H3 can regulate gene transcription^33^. We measured the pan-trimethyllysine level of mouse retina at P0 (postnatal day 0) and P10 by Western blotting. The pan-trimethyllysine levels increased about 20–30% in *rd1* mice than *wt* controls at P0 and P10 (Fig. 1a, b). To determine the lysine trimethylation sites, we applied tandem mass tags (TMT) labeling and lysine trimethylation affinity enrichment, followed by high-resolution liquid chromatography and mass spectrometry (LC-MS/MS) to P10 retinas (Fig. 1c). We had identified 6 sites in histone H3 that contain higher levels of lysine trimethylation in *rd1* retina. These sites included H3K27, H3K36, H3.1K27, H3.1K36, H3.1K37, and H3.1K79 (Table 1). Mammalian histone H3 has three major variants (H3.1, H3.2, and H3.3) and they have very similar amino acid sequences^34^. To further confirm this result, we assessed the global H3K27me3 level using EpiQuik global H3K27me3 quantification kit (Colorimetric), and found that H3K27me3 consistently increases in *rd1* retina at P10 (Fig. 1d). These results suggested that histone H3 lysine trimethylation probably is involved in the retinal degeneration.

**Table 1:** Identified sites of lysine trimethylation of histone H3 in *rd1* retina

Protein	Protein names	Position	Modified sequence	*rd1*/*wt* Ratio
F8WI35	Histone H3	27	_K(tr)SAPSTGGVK_	1.718832
F8WI35	Histone H3	36	_SAPSTGGVK(tr)KPHR_	2.805843
P68433	Histone H3.1	27	_K(tr)SAPATGGVK_	1.5685
P68433	Histone H3.1	36	_SAPATGGVK(tr)KPHR_	2.091536
P68433	Histone H3.1	37	_K(tr)SAPATGGVK(tr)KPHR_	2.103125
P68433	Histone H3.1	79	_EIAQDFK(tr)TDLR_	1.401175

### DZNep inhibits retinal degeneration and improves visual function of *rd1* mice

In *rd1* mice, rod loss begins at P8, reaches its death peak at about P14, and nearly all rods die before P21. Cone cells can survive for another 1–2 months^2, 5^. To examine whether histone H3 lysine trimethylation affects the survival of photoreceptors, we performed subretinal injection of DZNep into *wt* and *rd1* eyes. DZNep is an inhibitor of S-adenosylmethionine-dependent methytransferase^35^, and can reduce the protein levels of PRC2 componets^36^, and induce apoptosis in dividing cancer cells but not in normal cells^36^. However, it has not been reported whether DZNep has any effects on post-mitotic dying neurons.

**Fig. 2:**
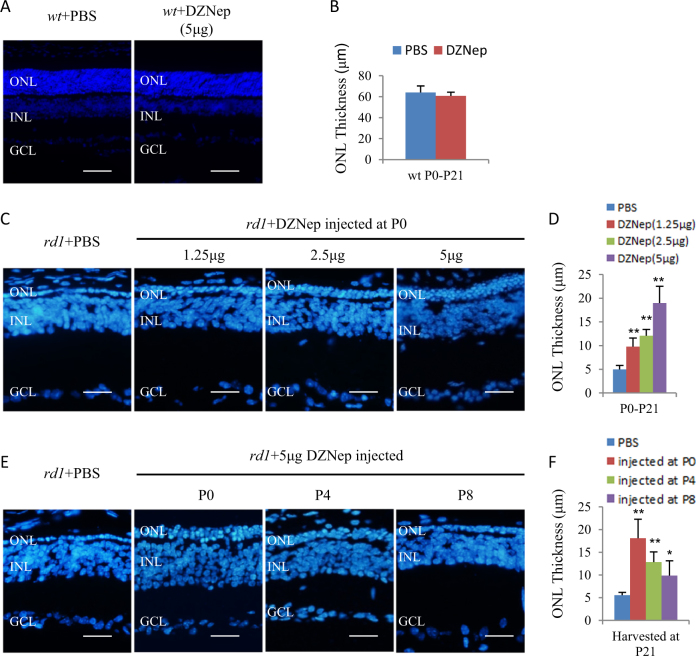
The effects of subretinal injection of DZNep on the thickness of ONL of *wt* and *rd1* mice. **a**, **b** PBS and 5 μg DZNep were injected into the subretinal space of *wt* mice at P0. The retinas were harvested at P21. Horizontal sections were stained for nuclear (DAPI, blue, **a**) and the ONL thickness was measured (**b**). **c**, **d** PBS and indicated dosages of DZNep were injected into the subretinal space of *rd1* mice at P0. The retinas were harvested at P21. Horizontal sections were stained for nuclear (DAPI, blue, **c**) and the ONL thickness was measured (**d**). **e**, **f** PBS was injected at P0, and 5 μg DZNep was injected into the subretinal space of *rd1* mice at indicated ages. The retinas were harvested at P21. Horizontal sections were stained for nuclear (DAPI, blue, **e**) and the ONL thickness was measured (**f**). Scale bar is 50 μm. Error bars represent SD of measurements from at least three animals and asterisks indicate significant differences between *rd1* retinas injected with PBS and DZNep of indicated dosages or ages. (*p < 0.05, **p < 0.01, one-way ANOVA followed by Bonferroni correction). ONL outer nuclear layer, INL inner nuclear layer, GCL ganglion cell layer

**Fig. 3:**
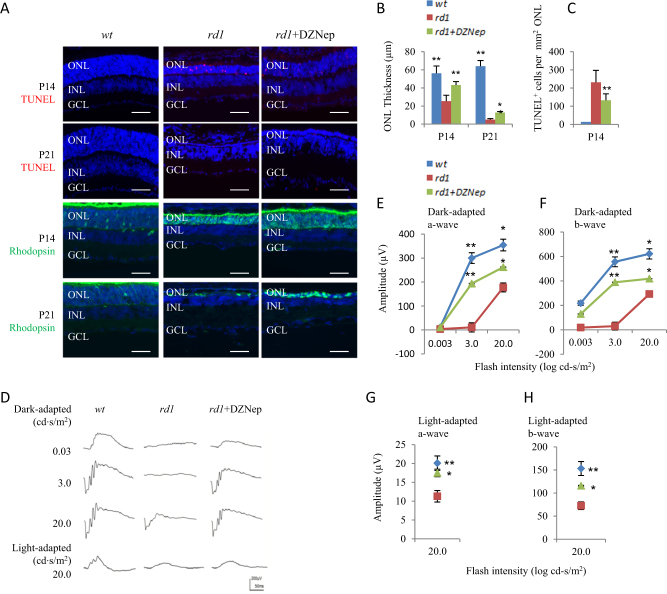
DZNep delayed the photoreceptor degeneration of *rd1* mice. 5 μg DZNep was injected into the subretinal space of *rd1* mice at P0. The retinas were harvested at P14 and P21. **a** Horizontal sections of *wt*, *rd1*, *rd1*/DZNep retinas were stained for nuclear (DAPI, blue), cell death (TUNEL, red), and rod photoreceptors (rhodopsin, green). **b** The ONL thickness of *wt*, *rd1*, and *rd1*/DZNep retinas at P14 and P21. **c** The TUNEL^+^ cells of *wt*, *rd1*, *rd1*/DZNep retinas at P14. **d**–**h** Retinal functions were evaluated by ERG at 14 days after DZNep treatment. Representative ERG responses in the *wt*, *rd1*, rd1/DZNEp retinas at P14 were shown in **d**, the ERG amplitude versus flash intensity for dark-adapted a-wave were in **e**, dark-adapted b-wave in **f**, light-adapted a-wave in **g**, and light-adapted b-wave in **h**. Scale bar is 50 μm. Error bars represent SD in **b** and **c**, and SEM in **e**–**h**, of measurements from at least three animals and asterisks indicate significant differences between *rd1* retinas and other two groups (*p < 0.05, **p < 0.01, one-way ANOVA followed by Bonferroni correction). ONL outer nuclear layer, INL inner nuclear layer, GCL ganglion cell layer

We injected three dosages (1.25 μg, 2.5 μg, and 5 μg) of DZNep at three time points (P0, P4, and P8). After injection, retinas were collected and analyzed at P21. Histological analysis showed no effects of DZNep on the thickness of outer nuclear layer (ONL) of *wt* retinas (Fig. 2a, b), whereas DZNep protected rods in a dose and time-dependent manner (Fig. 2c–f). Of the three dosages injected at P0, 5 μg had the most protective effects (Fig. 2c, d). Of three time points injected with 5 μg DZNep, the injection at P0 had the most protective effects (Fig. 2e, f). Therefore, injection of 5 μg DZNep at P0 was used for all the subsequent experiments and injected retinas were analyzed at P14 and P21. At P14, DZNep treatment increased the ONL thickness of *rd1* retinas from 25.38 ± 6.48 μm to 43.21 ± 3.78 μm (Fig. 3a, b). At P21, there was only one row of photoreceptors left (4.91 ± 1.15 μm) in *rd1* retinas, but in DZNep-treated *rd1* retinas there were at least three rows of photoreceptors left (12.73 ± 1.12 μm) (Fig. 3a, b). TUNEL labeling indicated that DZNep reduced cell death significantly, from 231.5 ± 61.6 cells/mm^2^ ONL to 133 ± 35.2 cells/mm^2^ ONL (Fig. 3a, c) in P14 *rd1* retinas. No TUNEL^+^ cells were detected in the ONL of rd1 retinas at P21 (Fig. 3a). Immunostaining confirmed that the survived cells at ONL were rhodopsin^+^ rods (Fig. 3a).

To test whether the survived rods had visual function, both dark- and light-adapted electroretinogram (ERG) were performed at P14 and P21 after DZNep treatment. The amplitudes of the dark-adapted ERG a- and b-wave, as well as light-adapted ERG a- and b-wave were both significantly preserved in the DZNep-treated *rd1* eyes, compared with that in PBS treated *rd1* eyes at P14 (Fig. 3d–h). However, the amplitudes were almost undetectable and did not differ significantly between DZNep-treated and PBS-treated *rd1* mice at P21 (data not shown). These results indicated that DZNep treatment can delay the rods death, and partially restore visual function of *rd1* mice at P14.

### DZNep inhibits Ezh2 protein level, H3K27me3 deposition, and calpain activity in ex vivo retinal explants of *rd1* mice

**Fig. 4:**
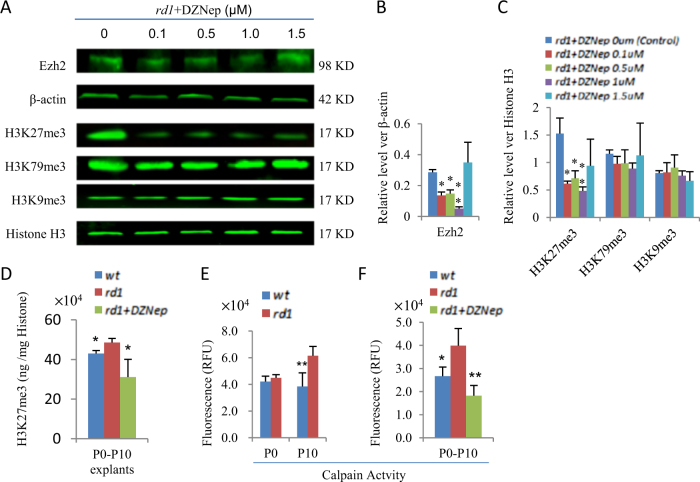
The effects of DZNep on the levels of Ezh2, H3K27me3, H3K79me3, H3K9me3, and Calpain activity of ex vivo retinal explant cultures from P0 to P10. **a** Representative Western blots of indicated proteins and treatments of *rd1* retinal explants. **b** The quantification of Ezh2 relative to β-actin in **a**. **c** The quantification of H3K27me3, H3K79me3, and H3K9me3 relative to histone H3 in **a**. **d** Direct measurement of H3K27me3 levels of retinas from *wt*, *rd1*, *rd1*/DZNep (1.0 μM) retinal explants. The Calpain activity in *wt* and *rd1* retinas at P0 and P10 (**e**), and in retinal explants of indicated genotypes and treatments cultured 10 days from P0 (**f**). Error bars represent SD of measurements from at least three animals and asterisks indicate significant differences between control group and other treatments in **b**, **c**; or between *rd1* retinas and *wt* or *rd1*/DZNep retinal explants in **d**, **f** (*p < 0.05, **p < 0.01, one-way ANOVA followed by Bonferroni correction)

To understand how DZNep protects *rd1* retina, we employed an ex vivo retinal explant system. Four different concentrations (0.1 μM, 0.5 μM, 1 μM, 1.5 μM) of DZNep had been tested. Western blotting confirmed that 0.1–1.0 μM DZNep can reduce the protein levels of both Ezh2 and H3K27me3 in the retinal explants, and 1.0 μM DZNep had the strongest effect (Fig. 4a–c). Interestingly, 1.5 μM DZNep had no major effects on the protein levels of Ezh2 and H3K27me3 (Fig. 4a–c). Therefore, we used 1 μM DZNep for the following ex vivo studies. Several studies indicated that DZNep may be a global inhibitor of methyltransferases^37^, not only suppressing Ezh2 and H3K27me3 levels^36^, but also inhibiting H3K9me3 and H3K79me3 formation^38, 39^. However, we had not found any effects of DZNep on H3K9me3 and H3K79me3 levels in our ex vivo retinal explant system (Fig. 4a, c).

Consistent with the in vivo results (Fig. 1d), H3K27me3 also increased in *rd1*ex vivo retinal explants, cultured from P0 to P10 (P0 + 10) (Fig. 4d), and DZNep treatment reduced it to *wt* level (Fig. 4d). Calpain is activated in *rd1* retina, and reducing calpain activity can delay photoreceptor degeneration^9, 40^. We measured calpain activities using a calpain activity assay kit. Consistent with previous reports, calpain activity was higher in *rd1* P10 retinas and (P0 + 10) retinal explants (Fig. 4e, f). DZNep treatment significantly suppressed its elevation in *rd1* P0 + 10 retinal explants (Fig. 4f). These results validated that ex vivo explants can represent the pathogenesis of in vivo *rd1* retina, like several previous studies^10, 19^, and suggested that DZNep can protect *rd1* retina by suppressing calpain activity and H3K27me3 deposition, but not H3K9me3 or H3K79me3.

### Ezh2/H3K27me3 interacts with PI3K-Akt pathway in *rd1* retina

**Fig. 5:**
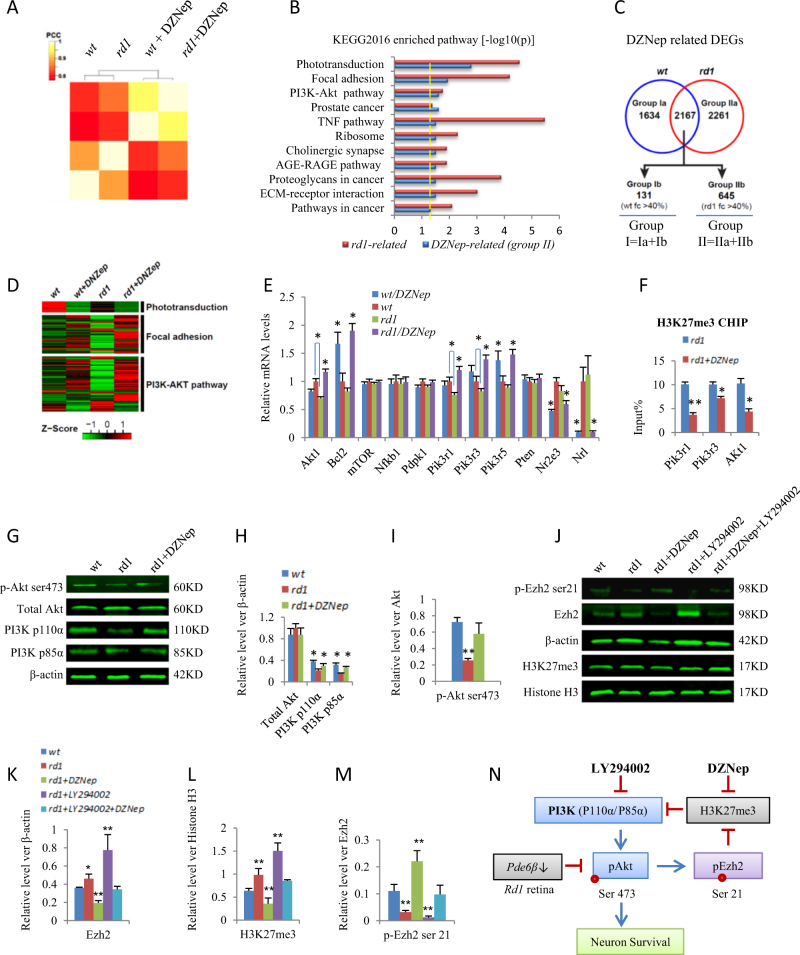
Ezh2/H3K27me3 inhibit PI3K-Akt pathway in rd1 retina. **a** Cluster analysis of coding gene expression profiles of *wt* and *rd1* mouse retinal explants with or without DZNep treatments. **b** Gene list enrichment analysis using KEGG 2016 datasets in Enrichr of *rd1*-regulated and DZNep-regulated *rd1* specific DEGs (−log10(p)). Dotted line indicates *p* < 0.05. **c** Comparison of DZNep targets between *wt* and *rd1* retinas. The genes in Group Ia and Ib are the DZNep targets only in *w*t retinal cells, whereas the genes in Group IIa and IIb are the DZNep targets only in *rd1* retinal cells. **d** Heatmap of top 3 DZNep-regulated *rd1* specific DEGs. **e** The relative mRNA levels of indicated DEGs and indicated treatment groups were analyzed by RT-PCR to validate the DEGs. **f** Chromatin immunoprecipitation (ChIP) using H3K27me3 antibody at the promoter of indicated genes and treatments in *rd1* retinal explant cultured from P0 to P10. The enrichment of H3K27me3 was quantified using q-PCR and normalized to input. **g** Representative Western blots of indicated proteins and treatments of *wt* and *rd1* retinal explants. **h** The quantification of total Akt, PI3K-P110α, PI3K-P85α protein level relative to β-actin in **g**. **i** The quantification of p-Akt ser473 relative to total Akt protein in **g**. **j** Representative Western blots of indicated proteins and treatments of *wt* and *rd1* retinal explants. **k** The quantification of p-Ezh2 relative to total Ezh2 in **i**. l The quantification of Ezh2 relative to β-actin in **i**. **m**. The quantification of H3K27me3 relative to Histone H3 in **i**. **n** An integrated PI3K-Akt-Ezh2-H3K27me3 feedback loop regulates photoreceptor survival in *rd1* retina. Error bars represent SD (in **h**, **i**, **k**–**m**) or SEM (in **e**, **f**) of measurements from at least three retinal explants and asterisks indicate significant differences between DZNep-treated and untreated groups in **e, f**, or between *wt* and *rd1* as indicated by square brackets in **e**, or between *rd1* group and other groups in **h**, **i**, **k–m** (*p < 0.05, **p < 0.01, one-way ANOVA followed by Bonferroni correction)

To understand why H3K27me3 increased in *rd1* retina and further explore the mechanism of DZNep protection on *rd1* photoreceptors, we performed RNA-sequencing on *wt* and *rd1* retinal explants with or without DZNep treatments (1.0 μM, P0 + 10). Consistent with previous reports^41^, average linkage hierarchical cluster analysis indicated that *rd1* retinas clearly separated from *wt* retinas, DZNep-treated retinas clearly separated from PBS-treated samples, suggesting that *rd1* and DZNep treatment changed transcriptome significantly (Fig. 5a). We analyzed differentially expression genes (DEGs), and identified 3052 genes as *rd1*-related DEGs (Supplementary Table 1). Gene enrichment analysis indicated that the most enriched pathways of *rd1*-related DEGs included TNF, phototransduction, focal adhesion, and PI3K-Akt pathways (Fig. 5b). ENCODE histone modification analysis at Enrichr indicated that the most common histone modification of these *rd1*-regulated genes is H3K27me3; supporting H3K27me3-mediated gene silencing plays a role in *rd1* pathogenesis (Supplementary Table 2). We identified 6062 DZNep-related DEGs, including 1634 genes unique for *wt* retinas (Group Ia), 2261 genes unique for *rd1* retinas (Group IIa), and 2167 genes for both (Fig. 5c). Among these 2167 shared genes, 131 genes were filtered to Group Ib since they had 40% more expression changes in *wt* than that in *rd1* retina, whereas 645 genes were in Group IIb as they had 40% more expression changes in *rd1* retina. We then combined Group Ia and Ib to Group I (1765 genes), and Group IIa and IIb to Group II (2906 genes), respectively (Supplementary Table 3, Fig. 5c), and perform pathway analysis. Interestingly, we did not find any enriched pathways in Group I, as all adjusted *p* value > 0.05 (Supplementary Table 4), thus consistent with our observation that 1.0 μM DZNep did not cause any changes in *wt* retina (Fig. 2a).

However, we identified 17 pathways in Group II (adjusted *p* < 0.05), suggesting that DZNep affects these pathways to delay *rd1* photoreceptors death. Interestingly, 11 of these 17 DZNep pathways are the same pathways that caused *rd1* phenotypes (Fig. 5b). These results indicated that DZNep synergistically acts on several molecular pathways that cause *rd1* photoreceptor degeneration, revealing the therapeutic potential of DZNep for RP treatment. Heatmap of top three DZNep pathways, including phototransduction, focal adhesion, and PI3K-Akt pathway, were shown in Fig. 5d. We then focused on PI3K-Akt pathway for further investigation, as focal adhesion pathway overlaps with this pathway, and it was reported that this survival pathway is inactivated in *rd1* retina during photoreceptor degeneration^42^. The PI3K pathway is crucial to many aspects of cell adhesion, cell growth and survival. Class I PI3Ks function as heterodimers with one of four catalytic p110 subunits (p110α, β, δ, or γ) and a regulatory subunit (p85α, p85β, p55γ, p101, or p84)^43, 44^. PI3K activation leads to the production of PIP3 which recruits Akt to the plasma membrane, where Akt gets phosphorylated and activated on Thr308 and Ser473 by Pdk1^45^ and mTORC2^46^, respectively. Pten can antagonize PI3K action by removing the 3-phosphate from PIP3^47^, and Pten expression is suppressed by NF-κB^48^.

RT-PCR results confirmed the expression changes of several PI3K-Akt pathway genes are consistent with RNA-sequencing data, such as *Akt1*, *Bcl2*, *mTOR*, *Nfkb1*, *Pik3r1*(encodes p85α), *Pik3r3*(encodes p55γ), *Pik3r5*(encodes p101), *Pdpk1*(encode Pdk1), and *Pten* (Fig. 5e). Comparing to *wt* retinas, *Akt1*, *Pik3r1*, and *Pik3r3* mRNA were significantly reduced in *rd1* retina, while the mRNA levels of *Bcl2*, *mTOR*, *Nfkb1*, *Pdpk1*, *Pik3r5*, and *Pten* were not different between *wt* and *rd1* retinas. This indicated that reduced PI3K regulatory subunits and Akt1 expression may contribute to the inactivation of Akt in *rd1* retina^42^. Consistent with the RT-PCR results (Fig. 5e), chromatin immunoprecipitation (CHIP) found that DZNep treatment significantly reduced H3K27me3 deposition at the promoter of *Akt1*, *Pik3r1*, and *Pik3r3* genes in *rd1* retina (Fig. 5f), thus de-repressed these genes expression. The total Akt (Akt1, Akt2, and Akt3) protein levels were similar between *wt*, *rd1*, and *rd1/*DZNep retinal explants, but its active form, phospho-Akt (Ser473), was much reduced in *rd1* retina, which was rescued by DZNep treatment (Fig. 5g–i). This result coincided with the RNA levels of *Pik3r1, Pik3r3* (Fig. 5e), and protein level of PI3K subunits p110α and p85α (Fig. 5g–h), indicating DZNep treatment de-repressed PI3K subunits expression and activated Akt in *rd1* retina.

It was reported that Akt can phosphorylate Ezh2 protein at serine 21 and suppresses its methyltransferase activity by impeding Ezh2 binding to histone H3^49^. Thus H3K27me3 level is inversely correlated with Akt activity, and explain why *rd1* retina has higher H3K27me3. Indeed, while Ezh2 protein and H3K27me3 increased in *rd1* retina, phospho-Ezh2 ser21 reduced significantly in *rd1* retina comparing to *wt* controls (Fig. 5j–m). DZNep treatment reduced Ezh2 expression in *rd1* retina (Figs. 4a, 5j, k), but also increased the phospho-Ezh2 ser21 level, which may result from de-repressing PI3K and activating Akt. Consistent with this notion, PI3K inhibitor LY294002 had opposite effects of DZNep on *rd1* retina, and thus reversed the effects of DZNep on Ezh2, H3K27me3, and phospho-Ezh2 ser21 levels (Fig. 5j–m). Thus Akt-mediated phosphorylation of Ezh2 at serine 21 and H3K27me3-mediated repression of PI3K subunits expression form a negative feedback loop and this network contributes to the photoreceptor survival in *rd1* retina (Fig. 5n).

We also looked some genes in the phototransduction pathway, DZNep treatment inhibit the expression of many photoreceptor genes, such as *Nrl* and *Nr2e3* (Fig. 5d, e), which may also contribute to the protection effects of DZNep on *rd1* photoreceptors. Similar to this phenomenon, Ezh1/2 double knockout in adult striatal medium spiny neurons (MSNs) and Purkinje cells also downregulated many identity genes of MSN or Purkinje cells^50^.

## Discussion

Dysregulation of DNA methylation^19^ and histone acetylation^10, 15, 51^ recently has been found to be involved in the pathogenesis of RP. In this study, we demonstrated that histone trimethylation also increases in *rd1* mice, suppression of Ezh2 and H3K27me3 by DZNep delays the retinal degeneration. RNA-sequencing and Western blotting revealed that DZNep may protect the photoreceptors by several mechanisms including inhibiting calpain activity, activating Akt pathway, and inhibiting photoreceptor differentiation.

For the first time, we found that pan-trimethyllysine of histones significantly increases in *rd1* retina, and identified 6 upregulated sites of histone H3 tri-methylation, including H3K27, H3K36, H3.1K27, H3.1K36, H3.1K37, and H3.1K79. In general H3K4me3, H3K79me3, and H3K36me3 are associated with transcriptional activation, while H3K27me3 and H3K9me3 have been linked to transcriptional repression^52^. H3K37me3 can also inhibit gene expression^53^. Thus *rd1* retina has upregulated both active and repressive histone methylation marks. While it is possible that the interplay of these histone marks can contribute to the retinal degeneration, we focused on the role of H3K27me3 in *rd1* retina in this study.

H3K27me3 is a repressive chromatin mark and mediates epigenetic silencing, which is catalyzed by Ezh2/Eed-containing PRC2^28^. Mouse tissue array staining indicates that H3K27me3 initially distribute throughout all retinal cells at E17.5, and from P1 onward, H3K27me3 mainly deposits in ganglion, amacrine, and horizontal cells, but low levels of H3K27me3 can still be detected in other cell types including rods^23^. These features of H3K27me3 deposition coincide with the expression pattern of Ezh2 in mouse retina. Ezh2 is dynamically expressed during retinal development, it is enriched in retinal progenitors and downregulated in differentiated cells, by P10 Ezh2 expression is largely restricted to the ganglion cell layer (GCL) and inner nuclear layer (INL), and then extinguished around P30^24, 27, 31^. Four studies used different mouse *Cre* strains to conditionally knock out *Ezh2* or *Eed* in mouse retina to investigate the role of H3K27me3 in retina^24, 27, 31, 54^. While knocking out *Ezh2* in post-mitotic retinal ganglion cells (RGCs) has no major phenotypes^27^, inactivation of *Ezh2* or *Eed* in retinal progenitors leads to abnormal differentiation of late-born retinal cells (rods, bipolar and Müller glial cells) in the early postnatal stages, and reduced overall eye size and ONL thickness at later postnatal stages, maybe due to reduced progenitor pool and/or enhanced cell death^24, 27, 31, 54^. Thus low level of H3K27me3 in *wt* progenitors may not favor the survival of photoreceptors later.

Surprisingly, in the present study we found that H3K27me3 increases in postnatal *rd1* mouse retina. As H3K27me3 may protect *wt* retinal progenitors and their progeny, we applied HMT inhibitor DZNep to test if H3K27me3 has a different role on the photoreceptors in postnatal *rd1* retinas. Subretinal injection of DZNep at P0 *rd1* mice significantly delayed rods death and improved their ERG responses. These results support the notion that elevated H3K27me3 is associated with photoreceptor death in *rd1* mice.

Similar to *Ezh2* knockout in post-mitotic mouse RGCs (but not in progenitors)^27^, subretinal injection of DZNep into P0 *wt* retinas has no major effects on the ONL thickness (Fig. 2a), and we did not find any enriched pathways of DZNep-related *wt* retina specific DEGs (Supplementary Table 4). The possible reason of this phenomenon is that in P0 *wt* retinas, H3K27me3 level is already low, DZNep treatment cannot reduce the H3K27me3 level further (Figs. 1d,4d) and induce any observable phenotypes.

It is intriguing that H3K27me3 has opposite effects on *wt* retinal progenitors and postnatal *rd1* photoreceptors, but similar phenomenon was reported in a number of studies. For instance, knocking out PRC1 component *Bmi1* in *wt* mice causes degeneration of cone photoreceptors^55^, but knocking out *Bmi1* in *rd1* mice strongly delays the rod degeneration^25^. Knocking out Ezh1/2 in MSNs and Purkinje cells lead to progressive neurodegeneration in *wt* mice^50^, but inhibiting Ezh2 rescued Purkinje cell degeneration in *Atm*^*−/−*^ mice^32^. Thus Ezh2 or H3K27me3 is important both for normal development and pathogenesis of neurodegeneration, and maintain the homeostasis of H3K27me is closely related to the survival of photoreceptor and other neurons.

To understand why H3K27me3 increases and how DZNep protects *rd1* photoreceptors, we used an ex vivo retinal explant system, which can represent the in vivo* rd1* pathogenesis in several previous studies^10, 19^. Although DZNep may be a global inhibitor of methyltransferases^37^, and can suppress H3K9me3 and H3K79me3 formation in several cancer cell types^38, 39^, we had not found any effects of DZNep on H3K9me3 and H3K79me3 in our ex vivo retinal explant system. Thus DZNep can protect *rd1* retina by suppressing calpain activity and H3K27me3 deposition, but not H3K9me3 or H3K79me3. RNA-sequencing and transcriptome analysis revealed that the protective effect of DZNep in *rd1* may be associated with Akt pathway and photoreceptor differentiation.

Ezh2 is the core component of PRC2, and its HMT enzyme activity is modulated by several kinases, including Akt, Atm, Cdk1, and Cdk2. While Cdk2-mediated Ezh2 phosphorylation on Thr350 increases H3K27 methylation^56^, Ezh2 phosphorylation on Ser21 by Akt^49^, or Ser734 by Atm^32^, or Thr487 by Cdk1^57^ prevent it. We confirmed previous study that PI3K-Akt pathway is inactivated in *rd1* retinal explants^42^, as phospho-Akt ser473 significantly reduced. Reduced Akt activity results in reduced phospho-Ezh2 ser21, enhanced PRC2 activity, and high levels of H3K27me3 in *rd1* retina. PI3K inhibitor LY294002 has opposite effects of DZNep on *rd1* retinas. Thus PI3K-Akt pathway and H3K27me3 forms a feedback loop in *rd1* retina (Fig. 5g–n). Our result in *rd1* mice is similar to the *Atm*^*−/−*^ mice, an animal model of ataxia-telangiectasia and cerebellum neurodegeneration^32^. H3K27me3 elevated in *Atm*^*−/−*^ mice due to reduced phospho-Ezh2 ser734. Lentiviral knockdown *Ezh2* rescued Purkinje cell degeneration and behavioral abnormalities in *Atm*^*−/−*^ mice^32^. Thus PRC2 hyperactivity may be a common mechanism to induce neurodegeneration, and a new therapeutic target for RP treatment, and need to be further explored in the future.

We also found that DZNep downregulated many photoreceptor genes such as *Nrl* and *Nr2e3* (Fig. 5e), similar to the Ezh1/2 mutant striatal MSNs and Purkinje cells, which also downregulated many identity genes of MSN or Purkinje neurons^50^. Likely dysregulation of these neuronal identity genes is secondary to the over-expression of some direct PRC2 targets, but the exact mechanism is still unknown^58^. Nrl acts as a cell fate switch during retinal development, photoreceptor precursors that turn on Nrl become rods, whereas those that do not become cones^59^. Targeted inactivation of *Nrl* via CRISPR/Cas9-mediated genome editing can rescue retinal rod and cone degeneration and restore visual function in four different mouse RP models (*rd1*, *rd10*, *Rho*^*−/−*^, *RHO*^*P347S*^)^60, 61^. Thus DZNep may protect rod photoreceptor by downregulation of *Nrl* and its downstream target *Nr2e3*.

In summary, we found that *rd1* retina has high levels trimethylation of several lysine sites of histone H3, including lysine 27 (H3K27me3), downregulating H3K27me3 with PRC2 inhibitor DZNep can delay the photoreceptor degeneration in *rd1* mice, which is related with multiple signaling pathways, including PI3K-Akt and rod differentiation pathways. To our knowledge, this is the first report that elevated H3K27me3 histone mark is involved in the pathogenesis of *rd1* retinal degeneration, and is a potential therapeutic target for RP treatment.

## Materials and methods

### Animals

The *Pde6b*^*rd1*^ (*rd1*) mice on *ICR* background were purchased from Taconic (Germantown, NY, USA) and housed in the Laboratory Animal Center of Sichuan University (Chengdu, China) with a normal 12-h/12-h light/dark schedule. Fresh water and rodent diet were available at all times. Age-matched *wt ICR* mice were used as control. All animal procedures were performed in accordance with the statement of the Association for Research in Vision and Ophthalmology (ARVO), and the protocol was approved by Ethics Committee of Animal Research of West China Hospital, Sichuan University.

### Ex vivo retinal explant cultures and DZNep or LY294002 treatment

Retinal explants obtained from newborn *rd1* and *wt* mice were cultured using a protocol from previously described methods^10, 19, 62^. Briefly, both eyes were enucleated and retinas were carefully peeled away from the retinal pigment epithelium, and radial cuts were made to flatten the retina. The flattened retina was transferred to the membrane of a Millicell insert (Millipore, PICM03050) with the photoreceptors facing down. The insert was placed into the wells of a 6-well plate (Costar 3516, Corning), each contained 1300 μl of retinal explant media, which was replaced every 2 days, and was maintained in a 37 °C incubator with 5% CO_2_ for 10 days. The retinal explant basal medium was serum-free and made from Neurobasal A, DMEM/F12 medium, and N2/B27 supplements (Life Technologies, USA). Cultured explants were either exposed to DZNep (Cayman Chemical, 13828, Ann Arbor, USA) treatment (0.1 μM, 0.5 μM, 1 μM, 1.5 μM), LY294002 (Selleckchem, S1105, Houston, TX, USA) treatment (20 μM), or kept as PBS-treated controls.

### Western blotting

Total Histones or protein were extracted from retinas as well as retinal explants using the total histone extraction kit (Epigentek, Farmingdale, NY, USA) or RIPA lysis buffer containing 1% proteases inhibitor and phosphatase inhibitors (Beyotime Institute of Biotechnology, Haimen, China) according to the manufacturer’s instructions. The protein concentration was determined with a bicinchoninic acid (BCA) assay kit (Beyotime). Then, 30 μg proteins was separated by 15% or 12% SDS-polyacrylamide gel and the separated proteins were transferred to polyvinylidene difluoride membranes (Millipore, Billerica, MA, USA). After blocking with 5% non-fat milk or 5% bovine serum albumin, the membranes were incubated with primary antibodies against Akt (1:1000, Cell Signaling Technology, 9272, Beverly, MA, USA), Phospho-Akt (Ser473) (1:1000, Cell Signaling Technology, 4058, Beverly, MA, USA), Ezh2 (1:1000, Cell Signaling Technology, 3147, Beverly, MA, USA), Phospho-Ezh2 (Ser21) (1:200, Abcam, ab84989, Cambridge, MA), H3K27me3 (1:1000, Cell Signaling Technology, Beverly, 9733, MA, USA), H3K9me3 (1:500, Millipore, 07-442, Billerica, MA, USA), H3K79me3 (1:1000, Abcam, ab2621, Cambridge, MA), Histone H3 (1:2000, Cell Signaling Technology, 4499, Beverly, MA, USA), Pan-trimethyllysine (1:1000, PTM-Biolabs, PTM-601, Hangzhou, China), PI3K p110α (1:1000, Cell Signaling Technology, 4249, Beverly, MA, USA), PI3K p85α (1:1000, Cell Signaling Technology, 13666, Beverly, MA, USA), and β-actin (1:2000; Abcam, ab6276, Cambridge, MA). After washing, the membrane was incubated with secondary antibody for 1 h. The images were captured on the Odyssey CLx Infrared Imaging System (LI-COR Biosciences, Lincoln, NE, USA). Western blotting bands were quantified using Odyssey CLx v2.1 software and normalizing to histone H3 (H3K27me3, H3K9me3, H3M29me3) or β-actin (Akt, Ezh2, PI3K p110α, PI3K p85α) as loading control, or Akt (pAkt ser473), and Ezh2 (p-Ezh2 ser 21).

### Quantification of histone lysine trimethylation

The quantification of histone lysine trimethylation in retinas were performed by PTM Biolabs (Hangzhou, China) with integrated approach involving TMT labeling, HPLC fractionation, lysine trimethylation affinity enrichment, mass spectrometry-based quantitative histone to quantify dynamic changes. Briefly, total histone was first extracted from retinas. After trypsin digestion, peptide was reconstituted in 0.5 M TEAB and processed according to the manufacturer’s protocol for 6-plex TMT kit (Thermo Scientific, Rockford, IL, USA). The sample was then fractionated into fractions by high pH reverse-phase HPLC using Agilent 300 Extend C18 column (5 μm particles, 4.6 mm ID, 250 mm length). To enrich lysine trimethylation peptides, tryptic peptides dissolved in NETN buffer were incubated with pre-washed antibody beads (PTM Biolabs) at 4 °C overnight with gentle shaking, and then washed and vacuum-dried. The resulting peptides were cleaned with C18 ZipTips (Millipore) according to the manufacturer’s instructions, followed by LC-MS/MS analysis by Q Exactive TMPlus hybrid quadrupole Orbitrap mass spectrometer (Thermo Fisher Scientific). The resulting MS/MS data was processed using MaxQuant with integrated Andromeda search engine (v.1.4.1.2). Tandem mass spectra were searched against UniProt mouse Histone database concatenated with reverse decoy database. Trypsin/P was specified as cleavage enzyme allowing up to 3 missing cleavages, 4 modifications per peptide, and 5 charges. Mass error was set to 10 ppm for precursor ions and 0.02 Da for fragment ions. Carbamidomethylation on Cys was specified as fixed modification and oxidation on Met, trimethylation on Lys, and acetylation on protein N-terminal were specified as variable modifications. False discovery rate (FDR) thresholds for protein, peptide, and modification site were specified at 1%. Minimum peptide length was set at 7. All the other parameters in MaxQuant were set to default values. The site localization probability was set as >0.75.

### Quantification of global tri-methyl histone H3K27

Total histones were extracted from retina of postnatal day 0 (P0) and P10 mice, as well as retinal explants using the total histone extraction kit (Epigentek, Farmingdale, NY, USA) according to the manufacturer’s Instructions. Quantification of trimethylated H3K27 was performed using the EpiQuik global tri-methyl histone H3-K27 quantification kit (Colorimetric). The optical density value of histone lysine methylation at 450 nm was normalized to the standard control protein. The amount of trimethylated H3K27 were calculated applying the following formulas: Amount (ng/mg protein) = (OD (sample−blank)/protein (μg) × slope) × 1000.

### Calpain activity assay

Calpain activity was measured with a Calpain Activity Assay kit (Biovision, Mountain View, CA) according to the instructions of the manufacturer. Retinas were collected from P0, P10 mice, and retinal explants. Detection of the cleavage substrate Ac-LLY-AFC was performed in a fluorometer (Perkin Elmer, Inc., Waltham, MA, USA) that was equipped with a 400-nm excitation filter and 505-nm emission filter.

### Subretinal injection of DZNep

One microliter of DZNep (1.25 μg/μl, 2.5 μg/μl, 5 μg/μl) in PBS (Cayman 13828, Ann Arbor, MI, USA) was injected into the subretinal space of one eye according to a method described previously^63^. The same volume of PBS was administrated as control. Briefly, newborn (P0), P4, and P8 *rd1* and *ICR wt* mouse pups were anesthetized by chilling on ice or anesthetized with the mixture of ketamine and xylazine, and a small incision was made in the eyelid with a 32-gauge needle to expose the eyeball. A small hole was made through the limbus with a 32-gauge needle, and then a blunt 33-gauge needle (Hamilton syringe) was inserted through the hole, avoiding damage to the lens and penetrating the retina under a dissecting microscope. The successful delivery of drug was confirmed by viewing fluorescein-positive subretinal blebs demarcating the retinal detachment in the injected retinal area. Such detachments usually can be resolved spontaneously within 1 to 2 days. All animals received antibiotic ointment to the conjunctival sac and were observed daily after operation.

### Histology immunostaining

Immunofluorescence was performed using previously described protocols^64^. Briefly, eyeballs were collected at P14 and P21 after DZNep injection, and were fixed by 4% paraformaldehyde in 0.1 M phosphate buffer saline (PBS, pH 7.4) at 4 °C for 60 min, dehydrated in 30% sucrose overnight, and frozen in OCT at −80 °C. Cryostat sections were cut at 5 μm and collected on glass slides and stored at −20 °C. For immunostaining, the retinal sections were dried at room temperature (RT) and incubated in blocking solution (5% normal donkey serum, 1% BSA, and 0.03% Triton X-100 in 1× PBS) for 1 h, then were incubated with primary antibodies (Rhodopsin 1:1000, Santa Cruz, SC-57433) at 4 °C overnight. Subsequently, slides were incubated with corresponding secondary antibodies, conjugated to Alexa Fluor 488 (Invitrogen, USA), for 1  h at RT in the dark. Finally, slides were counterstained with 4′6-diamidino-2-phenylindole (DAPI; Sigma Aldrich Corp.) and mounted with Mowiol mounting medium. The thickness of ONL was measured in four areas at a distance of 500–1000 μm from the edge of optic disc. At least four sections of each eye were measured and data from four sections were averaged for each eye.

### TUNEL staining

To determine whether cell death had occurred, the frozen sections of mice eyes with or without DZNep treatment at P14 and P21 were labeled by TdT-dUTP terminal nick-end labeling (TUNEL) with an apoptosis detection kit (In Situ Cell Death Detection Kit; Roche Diagnostics, Mannheim, Germany) according to the manufacturer’s instructions. Non-specific signals were detected by omission of the enzyme reaction. Stained sections and slides were analyzed using a Zeiss Axio Imager Z2 fluorescence microscope, and Nikon A1RMP confocal microscope. Image J 1.50b with cell counter plugin was used for cell counting following the online guide. The positive cells of TUNEL were counted manually. At least 2 images per section, 2 sections per retina, and 3 retinas from each group were counted. All images for cell-counting were captured under fluorescence microscope using a 20× objective lens.

### Electroretinogram (ERG)

Both dark- and light-adapted ERG (Diagnosys LLC, Lowell, MA) were recorded at the 14th and 21th day after DZNep treatment. The procedures were performed as previous described^64^. In brief, mice were dark-adapted overnight and anesthetized with the mixture of ketamine and xylazine, and pupils were dilated with 0.5% tropicamide and 0.5% phenylephrine hydrochloride. Full-field ERG was recorded after inserting a ground electrode near the tail and a reference electrode on the back subcutaneously. A golden-ring electrode was gently positioned on the cornea. All procedures were performed under dim red light. The amplitude of the a-wave was measured from the baseline to the peak of a-wave, and the b-wave was measured from the nadir of the a-wave to the apex of the b-wave peak.

### RNA-sequencing

Total RNAs were extracted from retinal explants using TRIzol reagents (Invitrogen, Carlsbad, CA) and treated with RNase-free DNase I (New England Biolabs, Beverly, MA) to remove genomic DNA. The yield of total RNA was assessed using Nanodrop (Thermo). The cDNA libraries were prepared using the Illumina TruSeq RNA sample preparation kit and the qualities were assessed using an Agilent 2100 Bioanalyzer (Agilent Technologies, Palo Alto, CA). For sequencing, the cDNA libraries were loaded on an Illumina HiSeq 2500 at Biomaker (Beijing, China). The raw sequence reads in fastq format were processed and analyzed as previously reported^65, 66^. Briefly, the sequencing quality was first assessed using FastQC and poor quality 5′ end reads were trimmed using a Perl script and then mapped onto mouse genome (mm9) using Tophat2, allowing for up to two mismatches as default settings. Reads were mapped onto multiple genomic locations were discarded and a custom R script was used to calculate the RPKM of each gene and obtain expression profile of each sample. The expression fold change of each coding gene of *rd1* retina compared to *wt* retina was calculated using the following formula: Fold change = (RPKM + 1)^*rd1*^ / (RPKM + 1)^*wt*^. The genes, of which expression fold changes are >1.54 or <0.65, were selected as the *rd1*-related DEGs. The expression fold change of each coding gene after DZNep treatment was calculated using the following formula: Fold change = (RPKM + 1)^*+DZNep*^ / (RPKM + 1)^*−DZNep*^. The genes, of which expression fold changes are >1.54 or <0.65 upon drug treatment, were selected as the DZNep-related DEG. The function enrichment of DEG was performed using Enrichr^67, 68^, the pathways with adjusted *p* < 0.05 were chosen to report. The heatmap was generated using the R package of gplots and polished using Adobe Illustrator.

### RNA extraction, reverse transcription, and quantitative real-time PCR

Total RNA was extracted from retinal explants using Trizol Reagent (Invitrogen, Carlsbad, CA, USA) according to the manufacturer’s instructions. After quantifying by a Nanodrop Spectrophotometer (NanoDrop Technologies, Wilmington, DE, USA), cDNA was synthesized from 1 μg total RNA with PrimeScript RT reagent kit (Takara Biotechnology, Dalian, China). To quantify the cDNA, real-time PCR was performed on qTOWER 2.2 system (Analytik Jena, German). The PCR amplification was conducted in a volume of 20 μl using SsoFast^TM^EvaGreen^®^ Supermix (Bio-Rad Laboratories Inc., Hercules, CA, USA). The cycling protocol consisted of one cycle of 10 min at 95 °C followed by 40 cycles of 95 °C for 15 s and 60 °C for 1 min. To determine the mRNA expression, all samples were tested in duplicate and the average Ct values were used for quantification. The mRNA expression was normalized to the endogenous reference gene, glyceraldehyde 3-phosphate dehydrogenase (GAPDH). Relative quantification was achieved by using the 2^−ΔΔCt^ method as described previously^64^. Amplification of specific transcripts was confirmed by melting curve profiles at the end of each PCR. The primers sequences are listed in the Table 2.

**Table 2:** Sequences of primers for real-time PCR

Gene	Forward primer	Reverse primer
*Akt1*	5′−CACGGATACCATGAACGACG-3'	5′−GTTCTTGAGGAGGAAGTAGCGT−3′
*Akt1 promoter (ChIP)*	5′−GAAGAGCATCCGAGTGAGAAG−3′	5′−CTACACAGTTAGAGCCAGCAGG−3′
*Bcl-2*	5′−TGGGATGCCTTTGTGGAACTAT−3′	5′−AGAGACAGCCAGGAGAAATCAAAC−3′
*GAPDH*	5′−GCAAGGACACTGAGCAAGAGA−3′	5′−ATTCAAGAGAGTAGGGAGGGCT−3′
*mTOR*	5′−GAGAGGCTATCCGAGTGTTG−3′	5′−TGACTTGACTTGGACTCTGACA−3′
*Nfkb1*	5′−CAGAAAGATGACATCCAGAT−3′	5′−CAATGGCAAACTGTCTATGAAC−3′
*Nr2e3*	5′−GGTTGGGCCCAGCAACTTCT−3′	5′−CGCCACAGACACAGGCATAG−3′
*Nrl*	5′−GCTGCATTTTCACCGAATCT−3′	5′−GGTGGTTTGGGTTGTGGTAG−3′
*Pdpk1*	5′−*CTGTGAAGAGATGGAAGGGTA*−3′	5′−*ATTGTCGTAGTTGCCATAGCA*−3′
*PIK3R1*	5′−ACAGTAGGAGGAGGTTGGAAGA−3′	5′−TGCGTCAGCCACATCAAGTAT−3′
*PIK3R1 promoter (ChIP)*	5′−TGTGATGTGGGTAGCAGCCT−3′	5′−TGTGAGTTTTGGAAGCAGAGG−3′
*PIk3R3*	5′−AAGGACATCACAGGAAATACAA−3′	5′−ACTGTGTTGTTCTTGGGTATGA−3′
*PIK3R3 promoter (ChIP)*	5′−GACGATGGGGATCTTGTCTG−3′	5′−TCCTCACAAACCTCTTCACCT−3′
*PIK3R5*	5′−ACCCAAGAGGTCCAAGAGAA−3′	5′−CAGGAGGTCGGAGTCTGGTG−3′
*PTEN*	5′−CTTCCACAAACAGAACAAGATG−3′	5′−GCTCTATACTGCAAATGCTATC−3′

### Chromatin immunoprecipitation (ChIP)

The ChIP assay was performed using Magna ChIP^TM^ A/G Chromatin Immunoprecipitation Kit (Millipore Temecula, CA, USA) according to the manufacturer’s instructions. Briefly, retinal explants treated with or without DZNep were cut into small pieces and then cross-linked for 10–20 min by addition of formaldehyde to a final concentration of 1%. The cross-linking was stopped by adding 1/20 volume of 2.5 M glycine and lysed by SDS lysis buffer. Then cell lysate was sonicated and immunoprecipitated with antibodies specific to H3K27me3 (1:50, Cell Signaling Technology, 9733) or negative control IgG. After protein/DNA complexes were eluted, reverse cross-linked to free DNA and purified, the specific DNA fragments were quantified using real-time PCR and normalized to input from the same sample. The primer sequences for the promoters analyzed are provided in Table 2.

### Statistical analysis

All experiments were carried out with *n* = 3–15. Statistical analysis was undertaken using the GraphPad Prism software (GraphPad Prism Software, Inc., San Diego, CA, USA). The results were analyzed by one-way ANOVA followed by Bonferroni correction for multiple comparisons, unpaired Student’s *t*-test was used to assess significance between two groups. The threshold for significance was set at *p* < 0.05.

## Electronic supplementary material

Supplementary Table 1

Supplementary Table 2

Supplementary Table 3

Supplementary Table 4
